# Does maternal genetic liability to folate deficiency influence the risk of antiseizure medication-associated language impairment and autistic traits in children of women with epilepsy?

**DOI:** 10.1016/j.ajcnut.2023.05.023

**Published:** 2023-05-20

**Authors:** Elisabeth Synnøve Nilsen Husebye, Julia Romanowska, Anne-Lise Bjørke-Monsen, Nils Erik Gilhus, Kaja Selmer, Kristina Gervin, Bettina Riedel, Marte Helene Bjørk

**Affiliations:** 1 Department of Clinical Medicine, University of Bergen, Bergen, Norway; 2 Department of Medicine, Volda Hospital, Volda, Norway; 3 Department of Global Public Health and Primary Care, University of Bergen, Bergen, Norway; 4 Department of Medical Biochemistry and Pharmacology, Haukeland University Hospital, Bergen, Norway; 5 Department of Clinical Science, University of Bergen, Bergen, Norway; 6 Department of Neurology, Haukeland University Hospital, Bergen, Norway; 7 National Center for Epilepsy, Oslo University Hospital, Oslo, Norway; 8 Department of Research and Innovation, Division of Clinical Neuroscience, Oslo University Hospital, Oslo, Norway; 9 Pharmacoepidemiology and Drug Safety Research Group, Department of Pharmacy, School of Pharmacy, University of Oslo, Oslo, Norway

**Keywords:** MoBa, MBRN, autism spectrum disorder, language delay, polygenic risk score, folic acid, MTHFR

## Abstract

**Background:**

Prenatal exposure to antiseizure medication (ASM) may lead to low plasma folate concentrations and is associated with impaired neurodevelopment.

**Objectives:**

To examine whether maternal genetic liability to folate deficiency interacts with ASM-associated risk of language impairment and autistic traits in children of women with epilepsy.

**Methods:**

We included children of women with and without epilepsy and with available genetic data enrolled in the Norwegian Mother, Father, and Child Cohort Study. Information on ASM use, folic acid supplement use and dose, dietary folate intake, child autistic traits, and child language impairment was obtained from parent-reported questionnaires. Using logistic regression, we examined the interaction between prenatal ASM exposure and maternal genetic liability to folate deficiency expressed as polygenic risk score of low folate concentrations or maternal rs1801133 genotype (CC or CT/TT) on risk of language impairment or autistic traits.

**Results:**

We included 96 children of women with ASM-treated epilepsy, 131 children of women with ASM-untreated epilepsy, and 37,249 children of women without epilepsy. The polygenic risk score of low folate concentrations did not interact with the ASM-associated risk of language impairment or autistic traits in ASM-exposed children of women with epilepsy compared with ASM-unexposed children aged 1.5–8 y. ASM-exposed children had increased risk of adverse neurodevelopment regardless of maternal rs1801133 genotype {adjusted odds ratio [aOR] for language impairment aged 8 y was 2.88 [95% confidence interval (CI): 1.00, 8.26] if CC and aOR 2.88 [95% CI: 1.10, 7.53] if CT/TT genotypes}. In children of women without epilepsy aged 3 y, those with maternal rs1801133 CT/TT compared with CC genotype had increased risk of language impairment (aOR: 1.18; 95% CI: 1.05, 1.34).

**Conclusions:**

In this cohort of pregnant women reporting widespread use of folic acid supplements, maternal genetic liability to folate deficiency did not significantly influence the ASM-associated risk of impaired neurodevelopment.

## Introduction

Prenatal exposure to several antiseizure medications (ASMs) is associated with fetal growth restriction, congenital malformations, and impaired child neurodevelopment including language impairment and autism spectrum disorders (ASD) [1,2]. Various ASMs may interact with maternal folate uptake and metabolism [1,3], in addition to the pregnancy itself [4]. Long-term use of valproate, lamotrigine, oxcarbazepine, carbamazepine, phenytoin, and phenobarbital have all been associated with low folate concentrations [3,5], and subsequent increased risk of impaired neurodevelopment and preterm birth in some studies [6,7]. Because low folate concentrations during pregnancy are a risk factor for several child developmental disorders, women with epilepsy using ASM are recommended daily supplement of 0.4–5 mg of folic acid when planning pregnancy and during the first trimester [1,2,8,9].

Folate deficiency during pregnancy is associated with increased risk of congenital malformations, particularly neural tube defects and impaired neurodevelopment [4,10]. Folate plays a crucial role in 1-carbon transfer reactions involved in DNA and RNA synthesis, amino acid metabolism, and cellular methylation reactions [4,[11], [12], [13]]. These 1-carbon metabolism pathways support cellular functions and epigenetic regulation essential for normal fetal brain development [4,11,14]. Both low and high folate may alter DNA methylation patterns in the embryo and be harmful to fetal development [12,15,16]. Individual genetic variation in the form of single-nucleotide polymorphisms (SNPs) in genes regulating folate metabolism may affect plasma folate concentrations [14,16]. Polygenic risk score (PRS) summarizes the genetic risk of several SNPs associated with a specific phenotype [17]. PRS represents a proxy for the individual genetic liability to a phenotype, which is typically relevant for phenotypes associated with more than 1 genetic variation (polygenic etiology) [17]. The rs1801133 SNP in the *MTHFR* gene is the major genetic modifier of plasma folate concentrations [18]. This SNP (C>T) causes an amino acid substitution of alanine with valine and at least a 50% reduction in the enzymatic activity if both alleles are substituted (TT genotype) [14,19]. This leads to low plasma folate concentrations and high homocysteine concentrations unless folic acid supplementation is used [11,20]. The minor allele frequency (here allele T) is ∼33% in the European population [19,21]. The rs1801133 SNP and other SNPs affecting folate metabolism have been associated with increased risk of ASD and other neurodevelopmental disorders [14,16].

ASM-induced low plasma folate concentration has been suggested as a mechanism for ASM-associated impaired neurodevelopment [22,23]. We have previously found that periconceptional folic acid supplementation was associated with decreased risk of autistic traits and language impairment in ASM-exposed children of women with epilepsy enrolled in the Norwegian Mother, Father, and Child Cohort Study (MoBa) [[24], [25], [26]]. Periconceptional folic acid supplementation in women with epilepsy using ASM has been associated with better cognitive development in children at age 6 y [27], but not in all studies [28,29].

Despite increased knowledge regarding risk-reducing effects of periconceptional folic acid supplementation, the optimal dose of folic acid for women with ASM-treated epilepsy is unknown [2,30,31] and no recommendations exist on whether folate status should be monitored before and during pregnancy in women with epilepsy. We do not know whether children of women with ASM-treated epilepsy and with maternal genetic liability to folate deficiency during pregnancy are at higher risk of impaired neurodevelopment. The aim of this study was to examine if the maternal rs1801133 SNP in the *MTHFR* gene or PRS of low folate concentrations interact with the ASM-associated risk of language impairment and autistic traits in children of women with epilepsy. These results could influence future guidelines for individually adapted folic acid supplementation during pregnancy in women with epilepsy.

## Methods

### Study population

The data source was MoBa, a prospective, population-based pregnancy cohort study conducted by the Norwegian Institute of Public Health and linked to the mandatory Medical Birth Registry of Norway (MBRN) [32]. Pregnant women across Norway were invited to participate during the years 1999–2008 during gestational weeks 17–19, and 41% consented to participate [32]. Women answered 3 questionnaires during the pregnancy and 4 questionnaires after the child was born [32]. We used data regarding medical and social background, lifestyle exposures, medication, and folic acid supplement use during pregnancy collected in gestation weeks 17–19 and 30, and data regarding dietary folate intake and folic acid supplement dose from a food questionnaire in week 22 [30]. Parents-reported questionnaire-based data on child development were used from children aged 1.5, 3, 5, and 8 y [32]. Biological samples (whole blood and urine) were collected once during the pregnancy, in gestation weeks 17–19, and stored in the MoBa biobank [33]. MoBa Genetics is a research infrastructure within MoBa aiming to genotype all participants in MoBa [34]. The first data set of MoBa Genetics containing genetic data of a subsample of the cohort has been released for research [34].

**FIGURE 1:**
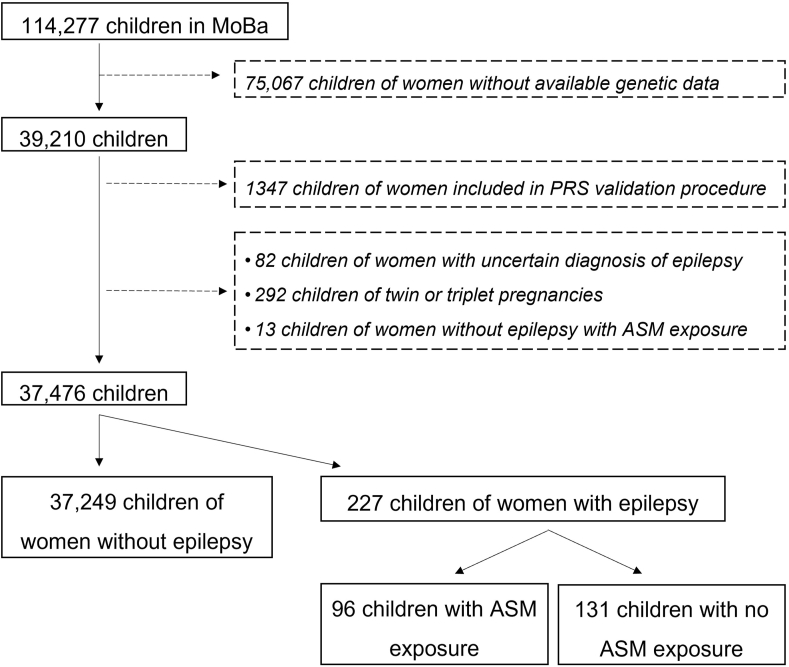
Flowchart of included and excluded cases. ASM, antiseizure medication; MoBa, The Norwegian Mother, Father, and Child Cohort Study; PRS, polygenic risk score.

We included women with and without epilepsy and their children aged 1.5–8 y enrolled in MoBa version 10, but with the exclusion of participants who had withdrawn from the MoBa cohort by September 2021 (*n* = 114,277). Children of women without available genetic data were excluded (*n* = 75,067). Children of women being part of the PRS validation procedure (see below) as well as children of twin or triplet pregnancies and ASM-exposed children of women without epilepsy were also excluded (*n* = 1734). The final study population consisted of singleton-born children of women with ASM-treated epilepsy (*n* = 96) and ASM-untreated epilepsy (*n* = 131), and a control group of children of women without epilepsy (*n* = 37,249; Figure 1).

### Epilepsy and ASM use

The maternal epilepsy cohort in MoBa has been described in detail [25,26,[35], [36], [37], [38]]. Women with epilepsy were identified based on self-reported MoBa questionnaires or diagnostic data from MBRN registered by a midwife or a physician. Data on ASM use during pregnancy were identified from MoBa questionnaires and MBRN. We categorized the women into ASM-treated and ASM-untreated epilepsy. The latter group reported present or previous epilepsy in MoBa, but no ASM use during the pregnancy. The maternal MoBa epilepsy cohort has previously been validated by examining ASM concentrations in maternal blood samples during pregnancy (*n* = 226) and in umbilical cord samples (*n* = 196) after birth, by examining medical records (*n* = 40), and by performing a retrospective survey (50% response rate) with questions including number of epileptic seizures during pregnancy and epilepsy type [35]. The validity was very good; the reported ASMs were detected in 93% of the biological samples, and 98% of the women that reported a diagnosis of epilepsy confirmed this in the retrospective validation survey [[35], [36], [37]].

### Autistic traits and language impairment

We collected information on autistic traits from the questionnaires at ages 3 and 8 y, and on language impairment from the questionnaires at ages 1.5, 3, 5, and 8 y. We examined autistic traits with the 40-item validated Social Communication Questionnaire [[39], [40], [41]]. Children with Social Communication Questionnaire score ≤11 points were defined as having autistic traits [39,40,42]. We examined language impairment at ages 1.5 and 3 y by using the 3- and 6-item communication scale of the validated The Ages and Stages Questionnaires (ASQ) [43], and by using a 1-item question on expressive language delay [44]. At age 1.5 y, children with a score of 1.5 SD or more below the mean score for the total MoBa population were defined as having language impairment [25,26,43]. At age 3 y, children with a score of 1.5 SD below the mean ASQ score for the total MoBa population or who talked in 2- to 3-word sentences or less were defined as having language impairment [26,42,45]. At age 5 y, we examined language impairment by using 3 different validated screening instruments from the 5-y questionnaire; a 7-item communication scale from the ASQ [43], a 13-item Speech and Language Assessment Scale [46], and the 20-item Norwegian instrument The Twenty Statements about Language-Related Difficulties (Language 20) [47]. Children filling the criteria in at least 1 test were defined as having language impairment; ASQ score of 1.5 SD or more below the mean score of the MoBa population, mean Speech and Language Assessment Scale score below 3, or Language 20 score of 31% or more of maximum [26,42,43,46,47]. At age 8 y, we used the 8-item semantic subscale of Language 20 with a cutoff for language impairment of 31% or more of maximum [26,42,47].

### Maternal folate intake and status

The women reported use of folic acid supplement from 4 wk before the pregnancy and during gestation weeks 0–4, 5–8, 9–12, 13+, and weeks 13–16, 17–20, 21–24, 25–28, and 29+. They also reported on the frequency of intake in gestation weeks 17–19 and 30; daily, 4–6 times per week, or 1–3 times per week. Folic acid supplement dose (μg/d) was reported in a 1-item question in week 22 in the FFQ. Dietary folate intake (μg/d) was estimated by MoBa based on data from the FFQ [48]. We defined periconceptional folic acid use as any use of a folic acid supplement from 4 wk before the pregnancy and/or during the first trimester.

For the PRS validation procedure described below, we accessed plasma folate concentrations of a subgroup of women without epilepsy (*n* = 2911) previously analyzed in a separate MoBa project [49]. In these women, plasma folate was determined by a *Lactobacillus casei* microbiological assay [49]. We have previously accessed and analyzed maternal blood samples from singleton pregnancies in a subsample of the group of women with ASM-treated epilepsy in the maternal epilepsy cohort in MoBa (*n* = 227) [38,42]. In these samples, folate metabolites were analyzed at Bevital Laboratory, Bergen, by using LC-MS/MS methods, which allow correction for folate degradation that occurs in samples kept at room temperature [50]. The sum of the concentrations of the folate metabolites 5-methyltetrahydrofolate (mTHF) and 4-alfa-hydroxy-5-methyltetrahydrofolate (hmTHF) was used as a proxy for total maternal folate concentration, as described previously [38,42]. We did not have access to data on folate concentrations in women with ASM-untreated epilepsy.

### Calculation and validation of PRS

Preprocessing of the genetic data was done with PLINK 1.9 [51]. We calculated PRS of low folate concentrations by using genome-wide genotype data from MoBa Genetics as the target data and summary statistics from an Irish genome-wide association study of serum folate concentrations (*n* = 2232) [18] as base data. We used genetic data from MoBa Genetics version 1.0, where quality control and imputation had been performed by MoBa [34]. We filtered the SNPs from the summary statistics to retain only those that were highly associated with the phenotype (folate concentration), but only weakly correlated with one another (that is, in high linkage disequilibrium). The co-occurrence of SNPs between the 2 datasets was high (95% of all SNPs and 99% of those had minor allele frequency >5%). We then calculated the optimal PRS of low folate concentrations using an automated search for best fit with PRSice2 software [17]. The PRS automatically chosen by PRSice2 included only 2 SNPs (rs1801122 and rs7545014), both within the *MTHFR* gene region. To broaden the estimated genetic liability to low plasma folate concentrations, we manually extracted the second-best PRS as well. This PRS was chosen as the main PRS for our study because it was based on 76 SNPs involving multiple genes related to folate metabolism and function (Supplemental Figure 1 and Supplemental Table 1) [4]. We used the automatically calculated PRS based on 2 SNPs in a separate sensitivity analysis (see below). Both the main PRS and the PRS from the sensitivity analysis were validated by examining their ability to predict low folate concentrations by using linear regression models and nonparametric correlation. The validation procedure was performed in the subsample of women without epilepsy with previously analyzed serum folate concentrations in MoBa and with available data in MoBa Genetic version 1.0 (*n* = 1028). Twin or triplet pregnancies, ASM users among women without epilepsy, and folate concentrations <2.33 nmol/L were excluded from the validation procedure [49]. To adjust for any population stratification among the controls, we ran a principal component analysis on the entire genetic information and extracted 3 first principal components to use as covariates in the validation regression model. Another covariate was the time difference in weeks between the last folic acid supplement intake and gestation week 18.

### Maternal rs1801133 genotype

Because the maternal rs1801133 genotype (CC, CT, or TT) is the predominant genetic modifier of plasma folate concentrations, we wanted to examine this SNP in more detail. We categorized the maternal rs1801133 genotype as a separate dichotomous variable divided into CC (normal enzymatic activity) and CT or TT (reduced enzymatic activity) genotype. The CT and TT genotypes were grouped together to preserve power and because both genotypes are associated with reduced enzymatic activity [19].

### Statistical analyses

We used R version 4.2.1 to perform the statistical analyses. The outcome was a dichotomous categorical variable, thus we used logistic regression (glm() R function). Data from the various follow-up time points were examined as separate logistic regression models. We adjusted for siblings by using robust standard errors using the {lmtest} [52] and {sandwich} [53,54] R packages. We adjusted for the following covariates to separate the effects of supplement use and dietary folate intake from the maternal liability to folate deficiency: any periconceptional folic acid supplement use, dietary folate intake, and folic acid supplement dose. We used the following packages to create result tables and figures: {ggplot2} [55], {patchwork} [56], {gtsummary} [57], {gt} [58], and {flextable} [59].

In the main analyses, we used a logistic regression model including all the mentioned covariates and an interaction term between any prenatal ASM exposure because of maternal epilepsy and one of *1*) PRS of low folate concentrations, or *2*) maternal rs1801133 genotype (CC or CT/TT). The outcome was either language impairment or autistic traits in ASM-exposed children of women with epilepsy compared with ASM-unexposed children. To examine whether certain types of ASM treatment were associated with maternal genetic risk of low folate concentrations, we examined the correlation between maternal folate concentrations (mTHF plus hmTHF) and PRS of low folate concentrations in pregnant women with ASM-treated epilepsy stratified for type of ASM monotherapy by using nonparametric correlation analyses [Spearman’s rho (*r*)]. We performed a sensitivity analysis by repeating the main analyses using the automatically calculated PRS based on 2 SNPs.

We then estimated risk of language impairment or autistic traits stratified for maternal rs1801133 genotype (CC or CT/TT) in children of women with ASM-treated epilepsy compared with children of women without epilepsy, and, separately, in children of women with ASM-untreated epilepsy compared with children of women without epilepsy. We also examined risk of language impairment or autistic traits in children of women with maternal rs1801133 genotype CT/TT compared with genotype CC separately for each of the 3 study groups: ASM-exposed children of women with epilepsy, ASM-unexposed children of women with epilepsy, and children of women without epilepsy. Moreover, we examined the number of ASM-exposed children of women with epilepsy with and without language impairment or autistic traits stratified for type of ASM monotherapy and maternal rs1801133 genotype (CC or CT/TT). Two-sided *P* values <0.05 or 95% CI not including 1 were considered statistically significant.

### Ethics

The establishment of MoBa and the initial data collection were based on a license from the Norwegian Data Protection Agency and approval from the Regional Committees for Medical and Health Research Ethics. The MoBa cohort is currently regulated by the Norwegian Health Registry Act. All data and material in MoBa are collected with informed consent from the participants. The current study was approved by the Regional Committees for Medical and Health Research Ethics.

## Results

### PRS

Genomic positions and annotations of SNPs included in the PRS of low plasma folate concentrations are presented in Supplemental Figure 1. Allele frequencies and *P* values of Hardy-Weinberg Equilibrium tests for each included SNP are presented in Supplemental Table 1. Among the 1028 women without epilepsy who were part of the PRS validation procedure, the correlation between maternal plasma folate concentrations during gestation weeks 17–19 and the PRS of low folate concentrations was −0.04 [Spearman’s *r*; *P* value 0.170; Supplemental Figure 2]. The median maternal plasma folate concentration did not differ significantly between women with CC compared with CT/TT rs1801133 genotypes (median 9.20 nmol/L compared with 9.01 nmol/L; *P* value 0.123; Supplemental Figure 2).

### Study population

**FIGURE 2:**
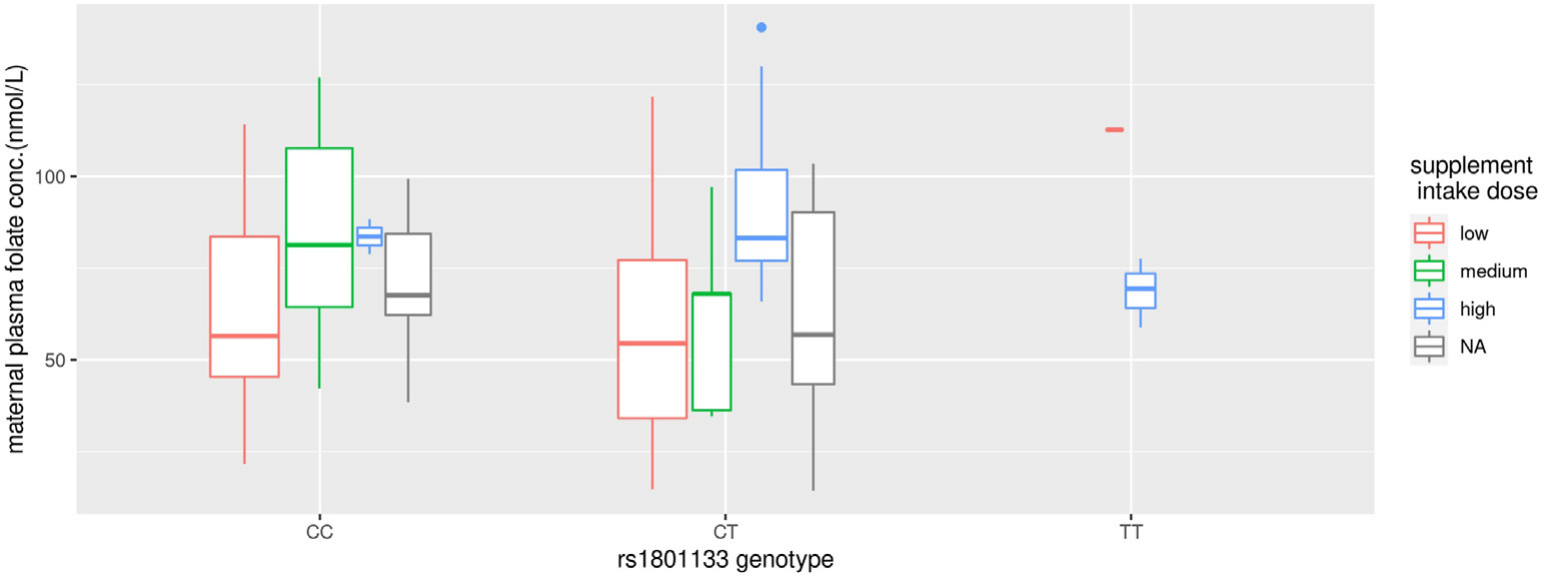
Maternal plasma folate concentrations (sum of concentrations of 5-methyltetrahydrofolate and 4-alfa-hydroxy-5-methyltetrahydrofolate) during gestation weeks 17–19 stratified by maternal rs1801133 genotype and folic acid supplement dose in women with antiseizure medication (ASM)-treated epilepsy (*n* = 80). The data are presented as boxplots with varying width: the middle line is the median, the box extends from the first to the third quartile, and the whiskers reach the largest value in the data, not larger than 1.5 × IQR; any data beyond these bounds are plotted as separate points. The width of the boxplot is proportional to the sample size. Folic acid supplement dose is defined as low dose if ≤0.4 mg/d, medium dose if 0.4 <1 mg/d, and high dose if ≥1 mg/d. NA, not applicable.

Because children of women being part of the validation procedure were excluded from the main analyses, the final study population consisted of 96 children of women with ASM-treated epilepsy, 131 children of women with ASM-untreated epilepsy, and a control group of 37,249 children of women without epilepsy (Figure 1). The most common fetal ASM exposures in utero were monotherapy with lamotrigine, carbamazepine, and valproate, as well as ASM polytherapy (Table 1). The questionnaire response rates decreased with increasing age of the child for all 3 groups (Table 1). A total of 80 children of women with ASM-treated epilepsy had available plasma folate concentrations (mTHF plus hmTHF) during pregnancy (Table 1). Any maternal folic acid supplement use from 4 wk before the pregnancy and until gestation week 20 was reported for 93 (97%) children of women with ASM-treated epilepsy, 111 (85%) children of women with ASM-untreated epilepsy, and 31,056 (86%) children of women without epilepsy. A total of 18,721 children had maternal rs1801133 genotype CC, whereas 18,755 children had maternal genotypes CT or TT (Supplemental Table 2), 3,199 (8.5%) of them having TT. Among pregnancies of women with ASM-treated epilepsy, the mean maternal folate concentration during gestation weeks 17–19 did not differ between pregnancies of women with CT/TT and CC genotypes (Supplemental Table 2), nor after stratification for the most common types of ASM monotherapy exposures (carbamazepine, lamotrigine, valproate; data not shown). The distributions of maternal plasma folate concentration during gestation weeks 17–19 stratified for maternal rs1801133 genotype and folic acid supplement dose are presented in Figure 2. We found no correlation between maternal plasma folate concentrations during gestation weeks 17–19 and PRS of low folate concentrations in pregnancies of women with ASM-treated epilepsy after stratification for the most common types of ASM monotherapy exposure (carbamazepine, *r* = −0.25, *P* value 0.309; lamotrigine, *r* = −0.08, *P* value 0.694; valproate, *r* = −0.28, *P* value 0.413; any ASM use, *r* = −0.21, *P* value 0.062; Supplemental Figure 3).

**TABLE 1:** Clinical characteristics of children of women with antiseizure medication (ASM)-treated and ASM-untreated epilepsy, and of children of women without epilepsy

	Children of women with ASM-treated epilepsy *N* = 96^1^	Children of women with ASM-untreated epilepsy *N* = 131^1^	Children of women without epilepsy *N* = 37,249^1^
ASM monotherapy exposure			
Valproate	12 (12%)	NA	NA
Carbamazepine	20 (21%)	NA	NA
Lamotrigine	32 (33%)	NA	NA
Levetiracetam	6 (6%)	NA	NA
Topiramate	<5	NA	NA
Oxcarbazepine	<5	NA	NA
Other	5 (5%)	NA	NA
ASM polytherapy exposure	16 (17%)	NA	NA
Maternal folate status during pregnancy			
Folic acid supplement dose (μg/d)	500 (211, 1100)	286 (0, 400)	200 (0, 400)
Missing	17	27	9022
Dietary folate intake (μg/d)	266 (217, 334)	270 (214, 332)	260 (208, 324)
Missing	10	10	4583
Periconceptional folic acid use^2^	78 (81%)	101 (77%)	28,610 (80%)
Missing	0	0	1352
Maternal plasma folate^3^ (nmol/L)	68 (51, 97)^4^	NA	NA
Missing	16	131	37,249
Questionnaire response rates			
Gestation week 17–19	96 (100%)	131 (100%)	35,897 (96%)
Missing	0	0	1352
Gestation week 30	90 (94%)	123 (94%)	34,230 (92%)
Missing	6	8	3019
Age 1.5 y	72 (75%)	96 (73%)	27,770 (75%)
Missing	24	35	9479
Age 3 y	60 (63%)	80 (61%)	22,219 (60%)
Missing	36	51	15,030
Age 5 y	38 (40%)	51 (39)	16,503 (44%)
Missing	58	80	20,746
Age 8 y	35 (36%)	61 (47%)	16,273 (44%)
Missing	61	70	20,976

Abbreviations: ASM, antiseizure medication; hmTHF, 4-alfa-hydroxy-5-methyltetrahydrofolate; mTHF, 5-methyltetrahydrofolat; NA, not applicable.

1 *n* (% of total) or median (IQR).

2 Any use during the period from 4 wk before the pregnancy and the first trimester.

3 Sum of mTHF and hmTHF.

4 *N* = 5 with plasma folate ≤28 nmol/L.

Interaction effects between prenatal ASM exposure due to maternal epilepsy and PRS of low folate concentrations or maternal rs1801133 genotype on language impairment and autistic traits

All adjusted odds ratios (aORs) from the logistic regression models with interaction terms are presented in Table 2 and Supplemental Tables 3 and 4. The interaction terms between any prenatal ASM use and either PRS of low folate concentrations or maternal rs1801133 genotype were not significant in any of the models for language impairment or autistic traits in ASM-exposed children of women with epilepsy compared with ASM-unexposed children aged 1.5, 5, and 8 y (all *P* values > 0.05; Table 2 and Supplemental Tables 3 and 4). This was also true for age 3 years, except for the interaction between ASM exposure and maternal rs1801133 genotype, which was significant (*P* value 1.34e-76; Supplemental Table 3). We found no significant interaction terms in the sensitivity analysis using the automatically calculated PRS based on 2 SNPs in the MTHFR gene (data not shown). In the interaction analyses, the covariates prenatal ASM exposure, dietary folate intake, and periconceptional folic acid supplement use were associated with risk of language impairment or autistic traits (Table 2 and Supplemental Tables 3 and 4).

**TABLE 2:** All estimates from the logistic regression models, including an interaction term between any prenatal ASM exposure due to maternal epilepsy and PRS of low folate concentrations. The outcome was language impairment in ASM-exposed children of women with epilepsy compared to ASM-unexposed children at ages 1.5, 3, 5, and 8 y. The aOR and *P* value of each covariate in the model is presented. Two-sided *P* values < 0.05 are marked with bold text.

Language impairment					
Covariates in the model		No language impairment, *n* (% of total)	Language impairment, *n* (% of total)	aOR (95% CI)	*P* value
Age 1.5 y					
Prenatal ASM exposure	No	19,768 (91)	2074 (10)	1.70 (0.83, 3.70)	0.190
	Yes	54 (86)	9 (14)		
Periconceptional folic acid use^1^	No	2496 (89)	305 (11)	**0.84 (0.74, 0.96)**	**0.010**
	Yes	17,326 (91)	1778 (9)		
Diet folate intake (μg/d)	Mean (SD)	277.1 (100.8)	267.0 (89.0)	**1.00 (1.00, 1.00)**	**<0.001**
Folic acid supplement dose (μg/d)	Mean (SD)	271.7 (272.5)	266.7 (273.9)	1.00 (1.00, 1.00)	0.590
PRS of low folate 2	Mean (SD)	-5.75e-05 (1.17e-03)	-2.79e-05 (1.20e-03)	1.00 (0.98, 1.10)	0.270
*Prenatal ASM exposure ∗ PRS of low folate*^3^				*1.10 (0.63, 2.00)*	*0.710*
Age 3 y					
Prenatal ASM exposure	No	17,270 (94)	1108 (6)	1.00 (0.31, 3.30)	0.980
	Yes	52 (95)	<5		
Periconceptional folic acid use 1	No	2127 (93)	160 (7)	**0.84 (0.70, 1.00)**	**0.047**
	Yes	15,195 (94)	951 (6)		
Dietary folate intake (μg/d)	Mean (SD)	276.3 (99.8)	271.5 (104.7)	1.00 (1.00, 1.00)	0.160
Folic acid supplement dose (μg/d)	Mean (SD)	274.8 (273.2)	268.2 (306.6)	1.00 (1.00, 1.00)	0.670
PRS of low folate 2	Mean (SD)	-5.54e-05 (1.18e-03)	-1.46e-05 (1.20e-03)	1.00 (0.98, 1.10)	0.270
*Prenatal ASM exposure ∗ PRS of low folate*^3^				*1.20 (0.37, 4.20)*	*0.730*
Age 5 y					
Prenatal ASM exposure	No	11,437 (80)	2957 (21)	**3.2 (1.50, 6.80)**	**0.001**
	Yes	22 (60)	15 (41)		
Periconceptional folic acid use 1	No	1067 (76)	332 (24)	**0.82 (0.72, 0.93)**	**0.003**
	Yes	10,392 (80)	2640 (20)		
Dietary folate intake (μg/d)	Mean (SD)	277.9 (98.7)	275.1 (106.6)	1.00 (1.00, 1.00)	0.200
Folic acid supplement dose (μg/d)	Mean (SD)	277.6 (274.0)	273.8 (284.9)	1.00 (1.00, 1.00)	0.530
PRS of low folate 2	Mean (SD)	-5.50e-05 (1.18e-03)	-7.17e-06 (1.20e-03)	1.00 (1.00, 1.00)	0.053
*Prenatal ASM exposure ∗ PRS of low folate*^3^				*1.30 (0.68, 2.40)*	*0.380*
Age 8 y					
Prenatal ASM exposure due to epilepsy	No	11,556 (82)	2494 (18)	**3.7 (1.70, 8.20)**	**0.001**
	Yes	21 (62)	13 (38)		
Periconceptional folic acid use 1	No	1366 (80)	333 (20)	**0.88 (0.77, 1.00)**	**0.045**
	Yes	10,211 (82)	2174 (18)		
Diet folate intake (μg/d)	Mean (SD)	275.7 (99.2)	274.5 (94.5)	1.00 (1.00, 1.00)	0.550
Folic acid supplement dose (μg/d)	Mean (SD)	272.7 (266.7)	272.5 (290.3)	1.00 (1.00, 1.00)	0.740
PRS of low folate 2	Mean (SD)	-6.05e-05 (1.19e-03)	-2.56e-05 (1.19e-03)	1.00 (0.99, 1.10)	0.210
*Prenatal ASM exposure ∗ PRS of low folate*^3^				*2.15 (0.87, 5.30)*	*0.088*

Abbreviations: ASM, antiseizure medication; aOR, adjusted odds ratio; NE, not estimable; NA, not applicable; PRS, polygenic risk score.

ASM-exposed children of women with epilepsy were compared to ASM-unexposed children using logistic regression models. Each age group was examined separately. Covariates in the adjusted models: periconceptional folic acid supplement intake (any intake during gestation week −4 to 12), dietary folate intake (μg/d), and folic acid supplement dose (μg/d).

1 Any use during the period from 4 wk before pregnancy and the first trimester.

2 aOR is given as change per 0.001 units of the PRS.

3 Interaction between prenatal ASM exposure due to maternal epilepsy and PRS of low folate concentrations. aOR is given as change per 0.001 units of the PRS.

### Maternal rs1801133 genotype and risk of language impairment and autistic traits

The aORs of language impairment or autistic traits were increased in children of women with ASM-treated epilepsy compared with children of women without epilepsy both for maternal rs1801133 CC and CT/TT genotypes at the various ages examined (TABLE 3, TABLE 4). The risk of language impairment or autistic traits in children of women with ASM-untreated epilepsy compared with children of women without epilepsy did not show any clear dependency on the maternal rs1801133 genotype (Supplemental Tables 5 and 6). In each of the 2 epilepsy groups, children with maternal rs1801133 genotypes CT/TT had no increased risk of language impairment or autistic traits compared with children with maternal genotype CC (Supplemental Tables 7 and 8). In children of women without epilepsy, children with maternal genotypes CT/TT had a slightly increased risk of language impairment compared with children with maternal genotype CC at age 3 y (aOR: 1.18; CI: 1.05, 1.34; Supplemental Table 7).

**TABLE 3:** Adjusted OR of language impairment in children of women with ASM-treated epilepsy compared with children of women without epilepsy stratified by maternal rs1801133 genotype

Maternal rs1801133 genotype	Maternal ASM-treated epilepsy	Child with language impairment, *n* (% of total)^1^		Crude OR (95% CI)	aOR (95% CI)
		No	Yes		
Age 1.5 y					
CC	No	12,272 (90)	1296 (10)	1.00	1.00
	Yes	26 (84)	5 (16)	1.82 (0.59, 5.63)	2.17 (0.69, 6.85)
CT/TT	No	12,325 (90)	1319 (10)	1.00	1.00
	Yes	35 (85)	6 (15)	1.60 (0.68, 3.78)	1.25 (0.46, 3.45)
Age 3 y					
CC	No	10,393 (94)	659 (6)	1.00	1.00
	Yes	27 (100)	0 (0)	0.00 (0.00, 0.00)	0.00 (0.00, 0.00)
CT/TT	No	10,372 (93)	759 (7)	1.00	1.00
	Yes	28 (85)	5 (15)	2.44 (0.91, 6.57)	1.62 (0.49, 5.42)
Age 5 y					
CC	No	6573 (80)	1668 (20)	1.00	1.00
	Yes	8 (57)	6 (43)	2.96 (0.88, 9.88)	2.97 (0.90, 9.83)
CT/TT	No	6523 (79)	1725 (21)	1.00	1.00
	Yes	15 (63)	9 (38)	2.27 (1.15, 4.46)	2.80 (1.41, 5.56)
Age 8 y					
CC	No	6597 (82)	1459 (18)	1.00	1.00
	Yes	12 (63)	7 (37)	2.64 (0.91, 7.66)	2.88 (1.00, 8.26)
CT/TT	No	6661 (82)	1446 (18)	1.00	1.00
	Yes	10 (63)	6 (38)	2.76 (1.10, 6.94)	2.88 (1.10, 7.53)

Abbreviation: ASM, antiseizure medication.

Children of women with ASM-treated epilepsy were compared to children of women without epilepsy stratified for maternal rs1801133 genotype by using multiple logistic regression models. Each age group was examined separately. Covariates in the adjusted models: periconceptional folic acid supplement intake (any intake during gestation week −4 to 12), dietary folate intake (μg/d), and folic acid supplement dose (μg/d).

1 Crude numbers.

**TABLE 4:** Adjusted OR of autistic traits in children of women with ASM-treated epilepsy compared with children of women without epilepsy stratified by maternal rs1801133 genotype

Maternal rs1801133 genotype	Maternal ASM-treated epilepsy	Child with autistictraits, *n* (% of total)^1^		Crude OR (95% CI)	aOR (95% CI)
		No	Yes		
Age 3 y					
CC	No	9829 (91)	1001 (9)	1.00	1.00
	Yes	21 (78)	6 (22)	2.81 (1.09, 7.23)	2.74 (1.03, 7.27)
CT/TT	No	9854 (90)	1059 (10)	1.00	1.00
	Yes	27 (82)	6 (18)	2.07 (0.82, 5.23)	1.71 (0.59, 4.97)
Age 8 y					
CC	No	7804 (98)	126 (2)	1.00	1.00
	Yes	18 (95)	<5	3.44 (0.45, 26.44)	4.16 (0.58, 29.68)
CT/TT	No	7866 (98)	150 (2)	1.00	1.00
	Yes	14 (88)	<5	7.49 (1.62, 34.58)	7.91 (1.74, 35.99)

Abbreviation: ASM, antiseizure medication.

Children of women with ASM-treated epilepsy were compared to children of women without epilepsy stratified for maternal rs1801133 genotype by using multiple logistic regression models. Each age group was examined separately. Covariates in the adjusted models: periconceptional folic acid supplement intake (any intake during gestation week −4 to 12), dietary folate intake (μg/d), and folic acid supplement dose (μg/d).

1 Crude numbers.

The number of children with language impairment and autistic traits stratified for the most common types of ASM exposure and maternal rs1801133 genotype are presented in Supplemental Table 9. For children exposed to carbamazepine monotherapy, all children with language impairment at ages 1.5–8 y had maternal rs1801133 genotypes CT or TT (Supplemental Table 9). Otherwise, there was no apparent association between language impairment or autistic traits and maternal rs1801133 genotype for children exposed to carbamazepine, lamotrigine, and valproate monotherapies (Supplemental Table 9).

## Discussion

In this study, we found that the maternal genetic liability to folate deficiency did not interact with the ASM-associated risk of language impairment or autistic traits in ASM-exposed children of women with epilepsy compared with ASM-unexposed children. We are not aware of previous studies examining the impact of maternal genetic liability to low folate concentrations and association to neurodevelopmental delay in children of women with epilepsy. By using a PRS of low folate concentrations based on 76 SNPs, as well as another PRS based on 2 SNPs within the *MTHFR* gene, and also the maternal rs1801133 genotype as a proxy of maternal genetic liability to folate deficiency, we included SNPs associated with different parts of folate metabolism and folate function [4,18]. This strategy takes into account combined SNP effects [20].

In the interaction analyses, the most important factors related to risk of language impairment and autistic traits in ASM-exposed children were ASM exposure as well as folic acid supplement use, in line with our previous findings [25,26]. Both these exposures may have overshadowed a potential negative effect of maternal genetic liability to folate deficiency. Folic acid supplement use was very common in our study population, and women with ASM-treated epilepsy reported higher doses of folic acid. Studies from the general population have shown that genetic risk of low folate concentration is counterbalanced by folic acid supplement use [11,20]. This is supported by 2 studies in nonpregnant adults with ASM-treated epilepsy. They revealed that the actual concentrations of folate and vitamin B12, but not the genetic variants of homocysteine metabolism predicted folate status measured as homocysteine concentrations [60]. Furthermore, homocysteine concentrations were reduced after vitamin B supplementation that included folic acid in adults with ASM-treated epilepsy [61]. We have previously shown the widespread use of folic acid and nonfolic acid vitamin B supplements in the epilepsy cohort in MoBa [38]. We adjusted for folic acid supplement intake, folic acid supplement dose, and dietary folate intake in all of our models, but the large number of women reporting folic acid supplement use in both the first and second trimesters still makes it difficult to estimate the genetic influence. This was reflected by the lack of correlation between the PRS of low folate concentrations and the maternal plasma folate concentrations during gestation weeks 17–19 in the validation procedure. There were no available data on maternal plasma folate concentrations during the periconceptional period in MoBa. This period of pregnancy has been shown to be vulnerable to neurodevelopmental impairment if no maternal folic acid supplement is used in children of women with ASM-treated epilepsy [27]. Hence, our results strongly indicate that folic acid supplementation is a stronger predictor of folate concentrations than any genetic liability to low folate concentrations. Furthermore, the folate-lowering effect of chronic ASM use [3] seems to be a stronger predictor of risk of language impairment and autistic traits than the maternal genetic liability to folate deficiency because maternal rs1801133 genotype or PRS of low folate concentrations did not interact with the ASM-associated risk of adverse neurodevelopment in our data, except for 1 finding at age 3 y. The interaction between maternal rs1801133 and ASM exposure was significant with aOR >1, indicating stronger association between ASM exposure and language impairment at 3 y among children of mothers with CT/TT genotype. However, this finding needs to be interpreted with caution because the stratified analyses within the group of ASM-exposed children of women with epilepsy showed that there were no children with maternal CC genotype and language impairment at age 3 y. Also, no such association was found for the other age groups. This is supported by a study showing that the effect of valproate treatment on the rate of congenital malformations was much stronger than the influence of maternal rs1801133 genotypes CT/TT [62]. The mechanisms related to ASM-associated adverse neurodevelopment probably also involve nonvitamin B-dependent mechanisms [2,23].

The children with language impairment after prenatal carbamazepine monotherapy exposure all had maternal rs1801133 CT or TT genotype. Carbamazepine leads to low folate concentrations [3]. Despite widespread use of folic acid supplement in our study population, this finding may indicate that high-dose folic acid supplementation is particularly important for women with epilepsy using carbamazepine, but low numbers render low power and our results must be interpreted with caution. One previous study in women using ASM during pregnancy reported a 3–4 times higher risk of having a child with congenital malformation in women with the rs1801133 TT compared with CC genotype [63]. In studies from the general population, SNPs related to maternal folate metabolism, particularly rs1801133 genotype CT or TT, have been associated with increased risk of adverse neurodevelopment such as ASD, intellectual disability, and ADHD [16]. Although we could not confirm such associations, our findings support the importance of optimal folate status during pregnancy for women with ASM-treated epilepsy.

Strengths of our study include a prospective design as well as precise data on prenatal ASM exposure, folic acid supplement use, and data on dietary folate and folic acid supplement dose in women with and without epilepsy, and also available plasma folate concentrations. Follow-up data were prospectively collected several years after the exposure. The women in MoBa receive no information on biobank results and thus do not know their genetic risk for low folate concentrations. Limitations include loss to follow-up with increasing age in MoBa [64], and hence subgroups with a limited number of observations, making some results difficult to interpret. Data on autistic traits and language impairment were collected by the parents with cutoffs based on screening instruments and not based on a formal neurophysiological assessment. Folate concentrations were only measured once during pregnancy, and were only available in a random subsample of women with ASM-treated epilepsy and of women without epilepsy (PRS validation procedure). Moreover, the folate concentrations were measured with different methods in the validation group and the study group.

In conclusion, the maternal genetic liability to folate deficiency did not interact with the ASM-associated risk of language impairment and autistic traits in children of women with epilepsy. Widespread maternal use of folic acid supplements may counteract potential adverse effects of maternal genetic liability to folate deficiency in children of women with epilepsy. Our data support current guidelines recommending folic acid supplementation to women of reproductive age with ASM-treated epilepsy.

## Acknowledgements

The Norwegian Mother, Father, and Child Cohort Study is supported by the Norwegian Ministry of Health and Care Services and the Ministry of Education and Research. We are grateful to all the participating families in Norway who take part in this on-going cohort study. We thank the Norwegian Institute of Public Health (NIPH) for generating high-quality genomic data. This research is part of the HARVEST collaboration, supported by the Research Council of Norway (#229624). We also thank the NORMENT Centre for providing genotype data, funded by the Research Council of Norway (#223273), South East Norway Health Authorities, and Stiftelsen Kristian Gerhard Jebsen. We further thank the Center for Diabetes Research, the University of Bergen for providing genotype data and performing quality control and imputation of the data funded by the ERC AdG project SELECTionPREDISPOSED, Stiftelsen Kristian Gerhard Jebsen, Trond Mohn Foundation, the Research Council of Norway, the Novo Nordisk Foundation, the University of Bergen, and the Western Norway Health Authorities.

We acknowledge Ragnhild Lervik, Hege Marit Nyberg, and Åshild Wæhre for excellent laboratory assistance, and Gyri Veiby, MD, PhD, and Olav Spigset, MD, PhD, for contributing to data acquisition.

## Data Availability

Data from the Norwegian Mother, Father, and Child Cohort Study and the Medical Birth Registry of Norway used in this study are managed by the national health register holders in Norway (Norwegian Institute of Public Health). Data described in the manuscript, code book, and analytic code can be made available to researchers through an application at www.helsedata.no, provided approval from the Regional Committees for Medical and Health Research Ethics (REC), compliance with the EU General Data Protection Regulation (GDPR), and approval from the data owners. The consent given by the participants does not open for storage of data on an individual level in repositories or journals. The analytical code is available at https://github.com/jromanowska/ASM-genetic-liability-low-folate-interaction.

## Appendix A Supplementary data

Multimedia component1 Multimedia component1
