# Supplementary material for: Does maternal genetic liability to folate deficiency influence the risk of antiseizure medication-associated language impairment and autistic traits in children of women with epilepsy?
Source: Am J Clin Nutr. 2023 May 20;118(1):303–13. doi: 10.1016/j.ajcnut.2023.05.023 (PMC10375495; doi:10.1016/j.ajcnut.2023.05.023)
Supplement: Multimedia component1 [file mmc1.pdf]

Title: Does maternal genetic liability to folate deficiency influence the risk of antiseizure medication-associated language impairment and autistic traits in children of women with epilepsy?

First author: Elisabeth Synnøve Nilsen Husebye

Supplemental data

Contents

Figure 1. Genomic positions and annotations of single nucleotide polymorphisms (SNPs) included in the polygenetic risk score (PRS) of low plasma folate concentrations. 2

Figure 2. Correlation between smoothed maternal plasma folate concentrations (nmol/L) collected in gestational weeks 17-19 and polygenic risk score (PRS) of low folate concentrations (A) and maternal rs1801133 genotype (B) in women without epilepsy (n = 1,028) who were part of the validation procedure. 4

Figure 3. Correlation between maternal plasma folate concentrations (sum of concentrations of 5-methyltetrahydrofolate and 4-alfa-hydroxy-5-methyltetrahydrofolate, nmol/L) during gestation weeks 17-19 and polygenic risk score of low folate concentrations in pregnant women with antiseizure medication-treated epilepsy. 5

Table 1. Allele frequencies and p-values of Hardy-Weinberg Equilibrium (HWE) tests for each single nucleotide polymorphism (SNP) included in the polygenic risk score (PRS) of low folate concentrations. 6

Table 2. Clinical characteristics of children of women with and without epilepsy stratified for maternal rs1801133 genotype. 8

Table 3. All estimates from the logistic regression models including an interaction term between any prenatal antiseizure medication (ASM) exposure due to maternal epilepsy and maternal rs1801133 genotype. The outcome was language impairment in ASM-exposed children of women with epilepsy compared to ASM-unexposed children at ages 1.5, 3, 5, and 8 years. 9

Table 4. All estimates from the logistic regression models including either an interaction term between any prenatal ASM exposure due to maternal epilepsy and one of: (i) PRS of low folate concentrations, or (ii) maternal rs1801133 genotype. The outcome was autistic traits in ASM-exposed children of women with epilepsy compared to ASM-unexposed children at ages 3 and 8 years. 11

Table 5. Adjusted odds ratio (aOR) of language impairment in children of women with antiseizure medication (ASM)-untreated epilepsy compared to children of women without epilepsy stratified by maternal rs1801133 genotype. 13

Table 6. Adjusted odds ratio (aOR) of autistic traits in children of women with antiseizure medication (ASM)-untreated epilepsy compared to children of women without epilepsy stratified by maternal rs1801133 genotype. 14

Table 7. Adjusted odds ratio (aOR) of language impairment in children of women with maternal rs1801133 genotype CT/TT compared to genotype CC within each of the three study groups. 15

Table 8. Adjusted odds ratio (aOR) of autistic traits in children of women with maternal rs1801133 genotype CT/TT compared to genotype CC within each of the three study groups. 16

Table 9. Number of antiseizure medication (ASM)-exposed children of women with epilepsy with and without language impairment and autistic traits stratified by type of ASM exposure in monotherapy and maternal rs1801133 genotype. 17

Figure 1. Genomic positions and annotations of single nucleotide polymorphisms (SNPs) included in the polygenetic risk score (PRS) of low plasma folate concentrations.

Each chromosome panel is divided into four parts: the top part shows the names and positions of SNPs, directly underneath is a chromosome ideogram; under the ideogram, the light-blue-shaded area contains regulatory region annotations (if any) while the light-green-shaded area shows gene annotation per each SNP (if any). Following the figure is an explanatory table with chronological overview of the SNPs included (Table S1).

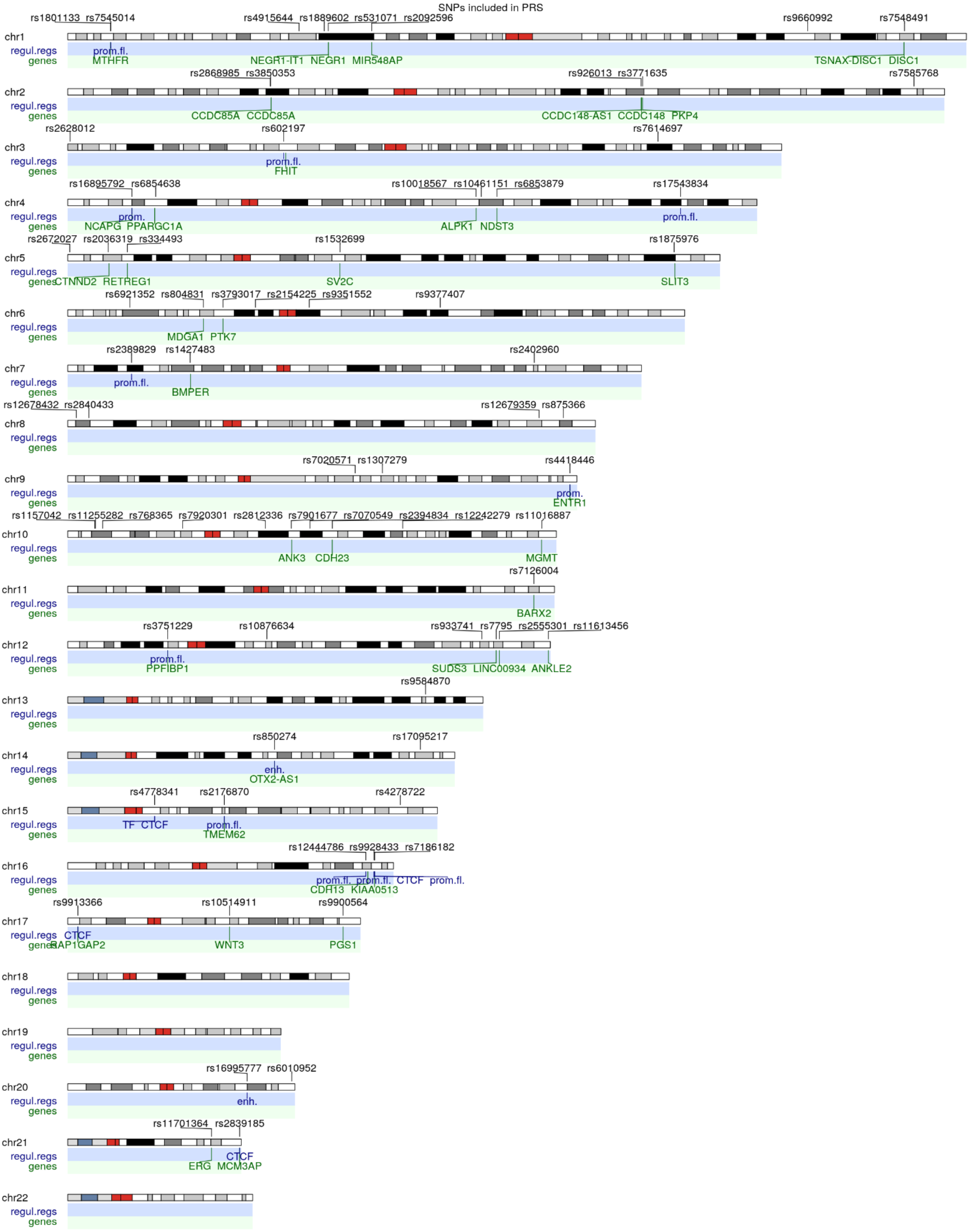
SNPs included in PRS
rs1801133 rs7545014 rs4915644 rs1889602 rs531071 rs2092596 rs9660992 rs7548491
chr1
regul.regs
genes
prom.fl.
MTHFR
NEGR1-IT1 NEGR1 MIR548AP
TSNAX-DISC1 DISC1
rs2868985 rs3850353 rs926013 rs3771635 rs7585768
chr2
CCDC85A CCDC85A
CCDC148-AS1 CCDC148 PKP4
rs2628012 rs602197 rs7614697
chr3
prom.fl.
FHIT
rs16895792 rs6854638 rs10018567 rs10461151 rs6853879 rs17543834
chr4
prom.
NCAPG PPARGC1A
ALPK1 NDST3
prom.fl.
rs2672027 rs2036319 rs334493 rs1532699 rs1875976
chr5
CTNND2 RETREG1
SV2C
SLIT3
rs6921352 rs804831 rs3793017 rs2154225 rs9351552 rs9377407
chr6
MDGA1 PTK7
rs2389829 rs1427483 rs2402960
chr7
prom.fl.
BMPER
rs12678432 rs2840433 rs12679359 rs875366
chr8
rs7020571 rs1307279 rs4418446
chr9
prom.
ENTR1
rs1157042 rs11255282 rs768365 rs7920301 rs2812336 rs7901677 rs7070549 rs2394834 rs12242279 rs11016887
chr10
ANK3 CDH23
MGMT
rs7126004
chr11
BARX2
rs3751229 rs10876634 rs933741 rs7795 rs2555301 rs11613456
chr12
prom.fl.
PPFIBP1
SUDS3 LINC00934 ANKLE2
rs9584870
chr13
rs850274 rs17095217
chr14
enh.
OTX2-AS1
rs4778341 rs2176870 rs4278722
chr15
TF CTCF
prom.fl.
TMEM62
rs12444786 rs9928433 rs7186182
chr16
prom.fl. prom.fl. CTCF prom.fl.
CDH13 KIAA0513
rs9913366 rs10514911 rs9900564
chr17
CTCF
RAP1GAP2
WNT3
PGS1
chr18
chr19
rs16995777 rs6010952
chr20
enh.
rs11701364 rs2839185
chr21
CTCF
ERG MCM3AP
chr22

Figure 2. Correlation between smoothed maternal plasma folate concentrations (nmol/L) collected in gestational weeks 17-19 and polygenic risk score (PRS) of low folate concentrations (A) and maternal rs1801133 genotype (B) in women without epilepsy (n = 1,028) who were part of the validation procedure. Correlation analysis was performed by using non-parametric correlation analysis with calculation of Spearman's rank correlation coefficient. p, p-value; rho, Spearman's rho.

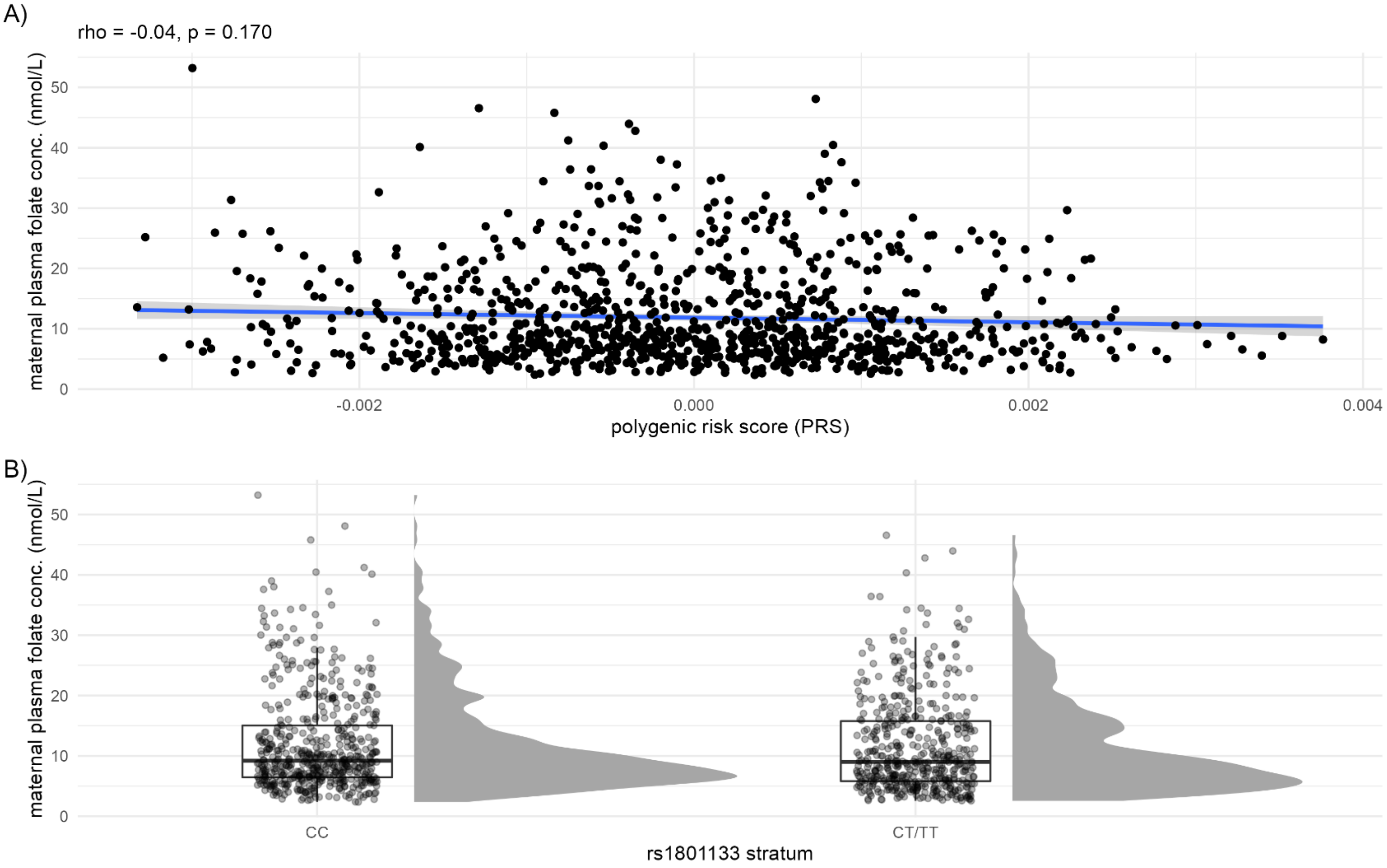

Figure 3. Correlation between maternal plasma folate concentrations (sum of concentrations of 5-methyltetrahydrofolate and 4-alfa-hydroxy-5-methyltetrahydrofolate, nmol/L) during gestation weeks 17-19 and polygenic risk score of low folate concentrations in pregnant women with antiseizure medication-treated epilepsy.

Data presented is unadjusted due to low numbers in each group. Correlation analysis was performed by using non-parametric correlation analysis with calculation of Spearman's rank correlation coefficient. ASM, antiseizure medication; p, p-value; PRS, polygenic risk score; rho, Spearman's rho.

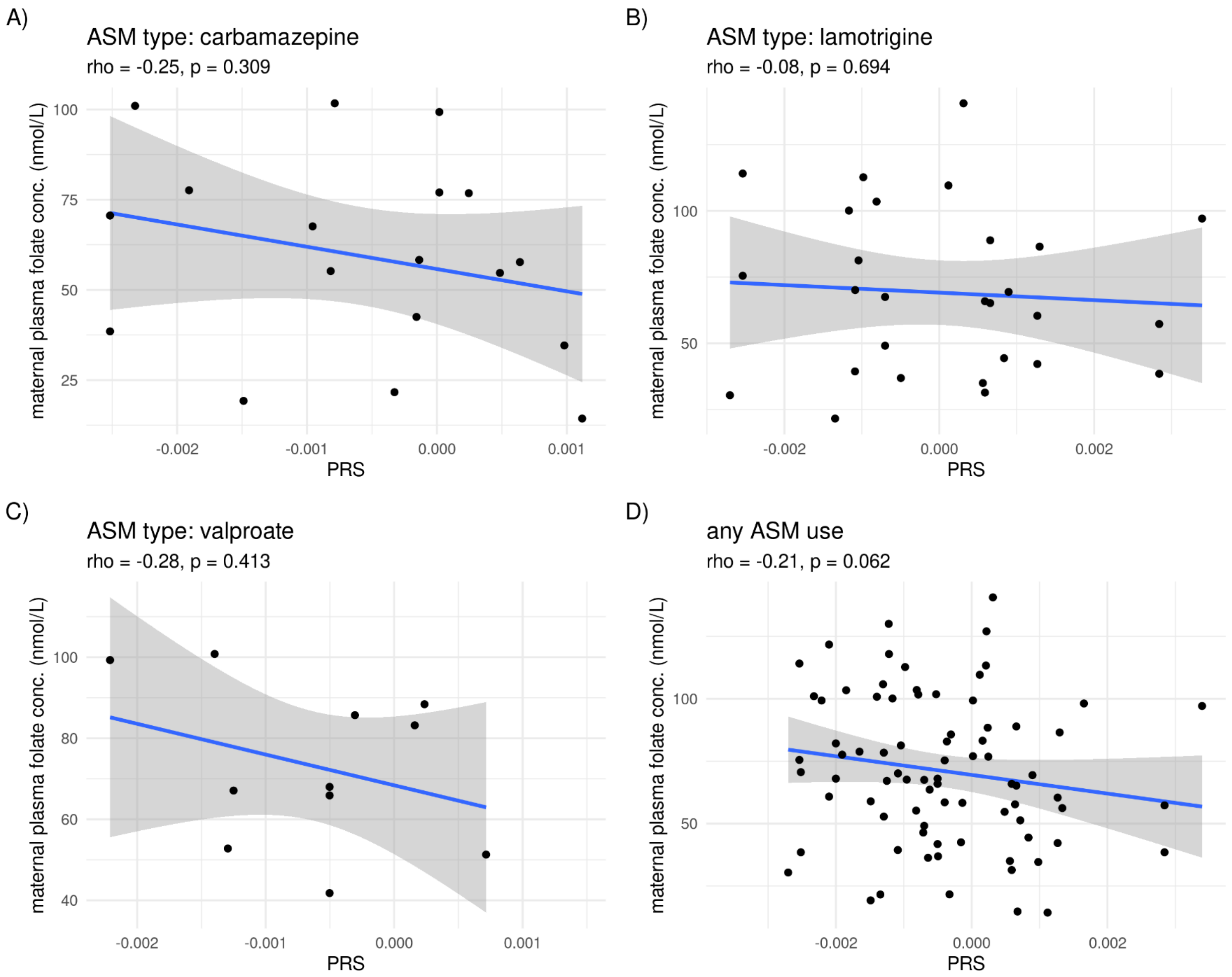

Table 1. Allele frequencies and p-values of Hardy-Weinberg Equilibrium (HWE) tests for each single nucleotide polymorphism (SNP) included in the polygenic risk score (PRS) of low folate concentrations.

SNP	Minor allele	Reference allele	Heterozygote freq.	HWE test p-value	MAF
rs1801133	T	C	106/429/577	0.41	0.29
rs7545014	T	C	201/543/368	0.49	0.42
rs4915644	G	A	34/319/759	0.29	0.17
rs1889602	G	T	82/458/572	0.40	0.28
rs531071	G	A	216/545/351	0.49	0.44
rs2092596	A	G	63/396/653	0.36	0.23
rs9660992	G	A	232/590/290	0.50	0.47
rs7548491	C	A	73/484/555	0.41	0.28
rs2868985	G	A	50/363/699	0.33	0.21
rs3850353	G	A	182/512/418	0.48	0.39
rs926013	C	A	23/264/825	0.24	0.14
rs3771635	C	T	89/437/586	0.40	0.28
rs7585768	T	C	230/569/313	0.50	0.46
rs2628012	G	A	8/171/933	0.15	0.08
rs602197	C	T	32/303/777	0.28	0.16
rs7614697	T	G	41/367/704	0.32	0.20
rs16895792	T	C	0/38/1074	0.03	0.02
rs6854638	T	C	135/513/464	0.46	0.35
rs10018567	A	G	3/67/1042	0.06	0.03
rs10461151	G	A	215/551/346	0.49	0.44
rs6853879	A	G	79/446/587	0.40	0.27
rs17543834	G	A	41/380/691	0.33	0.21
rs2672027	T	G	45/373/694	0.33	0.21
rs2036319	C	T	43/324/745	0.30	0.18
rs334493	C	T	6/181/925	0.16	0.09
rs1532699	G	T	147/539/426	0.47	0.37
rs1875976	C	T	167/527/418	0.47	0.39
rs6921352	G	T	287/531/294	0.50	0.50
rs804831	C	T	80/436/596	0.39	0.27
rs3793017	C	T	143/504/465	0.46	0.36
rs2154225	C	T	59/411/642	0.36	0.24
rs9351552	T	G	170/552/390	0.48	0.40
rs9377407	G	T	67/426/619	0.38	0.25
rs2389829	T	C	9/188/915	0.17	0.09
rs1427483	T	C	102/522/488	0.44	0.33
rs2402960	G	A	81/434/597	0.39	0.27
rs12678432	T	C	146/520/446	0.46	0.37
rs2840433	T	G	147/465/500	0.45	0.34
rs12679359	T	G	27/282/803	0.26	0.15
rs875366	T	C	89/470/553	0.41	0.29
rs7020571	G	T	188/523/401	0.48	0.40
rs1307279	C	T	60/365/687	0.34	0.22
rs4418446	A	G	73/421/618	0.38	0.25
rs1157042	A	G	145/497/470	0.46	0.35
rs11255282	C	T	115/499/498	0.44	0.33
rs768365	T	C	228/557/327	0.50	0.46
rs7920301	A	G	85/464/563	0.41	0.29
rs2812336	T	C	171/523/418	0.48	0.39
rs7901677	T	C	147/526/439	0.47	0.37
rs7070549	A	C	142/503/467	0.46	0.35
rs2394834	G	T	214/532/366	0.49	0.43
rs12242279	A	G	60/404/648	0.36	0.24

SNP	Minor allele	Reference allele	Heterozygote freq.	HWE test p-value	MAF
rs11016887	T	C	23/290/799	0.26	0.15
rs7126004	C	T	20/240/852	0.22	0.13
rs3751229	C	A	170/497/445	0.47	0.38
rs10876634	A	C	232/563/317	0.50	0.46
rs933741	C	T	44/295/773	0.29	0.17
rs7795	G	A	258/549/305	0.50	0.48
rs2555301	G	A	141/523/448	0.46	0.36
rs11613456	C	T	198/541/373	0.49	0.42
rs9584870	C	T	171/530/411	0.48	0.39
rs850274	A	G	185/504/423	0.48	0.39
rs17095217	A	G	145/513/454	0.46	0.36
rs4778341	A	G	41/288/783	0.28	0.17
rs2176870	G	A	44/345/723	0.31	0.19
rs4278722	G	T	88/416/608	0.39	0.27
rs12444786	A	G	35/300/777	0.28	0.17
rs9928433	G	A	43/348/721	0.31	0.20
rs7186182	C	A	5/114/993	0.11	0.06
rs9913366	C	T	207/575/330	0.49	0.44
rs10514911	A	G	11/213/888	0.19	0.11
rs9900564	G	A	125/514/473	0.45	0.34
rs16995777	A	G	1/91/1020	0.08	0.04
rs6010952	T	C	1/58/1053	0.05	0.03
rs11701364	A	G	31/266/815	0.25	0.15
rs2839185	A	G	30/329/753	0.29	0.17

Table 2. Clinical characteristics of children of women with and without epilepsy stratified for maternal rs1801133 genotype.

	**Children of women with rs1801133 genotype CC** N = 18,721[1]	**Children of women with rs1801133 genotype CT/TT** N = 18,755[1]
ASM monotherapy exposure		
Valproate	5 (<0.1%)	7 (<0.1%)
Carbamazepine	8 (<0.1%)	12 (<0.1%)
Lamotrigine	20 (0.1%)	12 (<0.1%)
Levetiracetam	<5	<5
Topiramate	<5	<5
Oxcarbazepine	0 (0%)	<5
Other	<5	<5
ASM polytherapy exposure	8 (<0.1%)	8 (<0.1%)
Maternal folate status during pregnancy		
Folic acid supplement dose (μg/day)	209 (0, 400)	200 (0, 400)
Missing	4,510	4,556
Dietary folate intake (μg/day)	260 (209, 325)	259 (207, 324)
Missing	2,323	2,280
Maternal plasma folate (nmol/L)[2]	69 (54, 91)	68 (41, 98)
Missing	18,681	18,715
Periconceptional folic acid use[3]	14,372 (80%)	14,417 (80%)
Missing	678	674
Children of women with ASM-treated epilepsy	47 (0.3%)	49 (0.3%)
Children of women with ASM-untreated epilepsy	63 (0.3%)	68 (0.4%)
Children of women without epilepsy	18,611 (99%)	18,638 (99%)

[1] n (% of total) or median (IQR)
[2] Sum of mTHF and hmTHF
[3] Any use during the period from four weeks before the pregnancy and the first trimester

Abbreviations: ASM, antiseizure medication; hmTHF, 4-alfa-hydroxy-5-methyltetrahydrofolate; IQR, interquartile range; mTHF, 5-methyltetrahydrofolate.

Table 3. All estimates from the logistic regression models including an interaction term between any prenatal antiseizure medication (ASM) exposure due to maternal epilepsy and maternal rs1801133 genotype. The outcome was language impairment in ASM-exposed children of women with epilepsy compared to ASM-unexposed children at ages 1.5, 3, 5, and 8 years.

The adjusted odds ratio (aOR) and p-value of each covariate in the model is presented in the table. Two-sided p-values <0.05 are marked with bold text.

Covariates in the model		Language impairment: No language impairment, n (% of total)	Language impairment: Language impairment, n (% of total)	aOR (95% CI)	P-value
**Age 1.5 years**					
Prenatal ASM exposure	No	19,768 (91)	2,074 (10)	2.20 (0.81 – 5.80)	0.190
	Yes	54 (86)	9 (14)		
Periconceptional folic acid use[1]	No	2,496 (89)	305 (11)	**0.84 (0.74 – 0.96)**	**0.010**
	Yes	17,326 (91)	1,778 (9)		
Dietary folate intake (µg/day)	Mean (SD)	277.1 (100.8)	267.0 (89.0)	**1.00 (1.00 – 1.00)**	**<0.001**
Folic acid supplement dose (µg/day)	Mean (SD)	271.7 (272.5)	266.7 (273.9)	1.00 (1.00 – 1.00)	0.650
Maternal rs1801133 genotype	CC	9,915 (90)	1,049 (10)	0.99 (0.90 – 1.10)	0.770
	CT/TT	9,907 (91)	1,034 (10)		
*Prenatal ASM exposure * rs1801133 genotype[2]*				*0.58 (0.14 – 2.50)*	*0.493*
**Age 3 years**					
Prenatal ASM exposure	No	17,270 (94)	1,108 (6)	**8.2e-06 (5.4e-06 – 1.2e-05)**	**<0.001**
	Yes	52 (95)	<5		
Periconceptional folic acid use[1]	No	2,127 (93)	160 (7)	**0.84 (0.70 – 1.00)**	**0.048**
	Yes	15,195 (94)	951 (6)		
Diet folate intake (µg/day)	Mean (SD)	276.3 (99.8)	271.5 (104.7)	1.00 (1.00 – 1.00)	0.160
Folic acid supplement dose (µg/day)	Mean (SD)	274.8 (273.2)	268.2 (306.6)	1.00 (1.00 – 1.00)	0.650
Maternal rs1801133 genotype	CC	8,690 (95)	509 (6)	**1.20 (1.00 – 1.30)**	**0.007**
	CT/TT	8,632 (94)	602 (7)		
*Prenatal ASM exposure * rs1801133 genotype[2]*				***2.1e+05 (5.9e+04 - 7.2e+05)***	***1.34e-76***
**Age 5 years**					
Prenatal ASM exposure	No	11,437 (80)	2,957 (21)	3.10 (1.10 – 9.00)	0.070
	Yes	22 (60)	15 (41)		
Periconceptional folic acid use[1]	No	1,067 (76)	332 (24)	**0.82 (0.72 – 0.93)**	**0.003**
	Yes	10,392 (80)	2,640 (20)		
Dietary folate intake (µg/day)	Mean (SD)	277.9 (98.7)	275.1 (106.6)	1.00 (1.00 – 1.00)	0.200
Folic acid supplement dose (µg/day)	Mean (SD)	277.6 (274.0)	273.8 (284.9)	1.00 (1.00 – 1.00)	0.560
Maternal rs1801133 genotype	CC	5,745 (80)	1,457 (20)	1.00 (0.96 – 1.10)	0.320
	CT/TT	5,714 (79)	1,515 (21)		
*Prenatal ASM exposure * rs1801133 genotype[2]*				*0.83 (0.21 – 3.20)*	*0.795*
**Age 8 years**					
	No	11,556 (82)	2,494 (18)	**2.90 (1.10 – 7.50)**	**0.048**

Prenatal ASM exposure due to epilepsy	Yes	21 (62)	13 (38)		
Periconceptional folic acid use[1]	No	1,366 (80)	333 (20)	**0.88 (0.77 – 1.00)**	**0.044**
	Yes	10,211 (82)	2,174 (18)		
Dietary folate intake (µg/day)	Mean (SD)	275.7 (99.2)	274.5 (94.5)	1.00 (1.00 – 1.00)	0.570
Folic acid supplement dose (µg/day)	Mean (SD)	272.7 (266.7)	272.5 (290.3)	1.00 (1.00 – 1.00)	0.890
Maternal rs1801133 genotype	CC	5,771 (82)	1,258 (18)	0.99 (0.90 – 1.10)	0.770
	CT/TT	5,806 (82)	1,249 (18)		
*Prenatal ASM exposure * rs1801133 genotype*[2]				*0.98 (0.24 – 3.90)*	*0.977*

[1] Any use during the period from four weeks before the pregnancy and the first trimester
[2] Interaction between prenatal ASM exposure due to maternal epilepsy and maternal rs1801133 genotype

ASM-exposed children of women with epilepsy were compared to ASM-unexposed children by using logistic regression models. Each age group was examined separately. Covariates in the adjusted models: periconceptional folic acid supplement intake (any intake during gestation week -4 to 12), dietary folate intake (µg/day), and folic acid supplement dose (µg/day).

Abbreviations: ASM, antiseizure medication; aOR, adjusted odds ratio; CI, confidence interval; NA, not applicable; NE, not estimable; PRS, polygenic risk score.

Table 4. All estimates from the logistic regression models including either an interaction term between any prenatal ASM exposure due to maternal epilepsy and one of: (i) PRS of low folate concentrations, or (ii) maternal rs1801133 genotype. The outcome was autistic traits in ASM-exposed children of women with epilepsy compared to ASM-unexposed children at ages 3 and 8 years.

The adjusted odds ratio (aOR) and p-value of each covariate in the model is presented in the table. Two-sided p-values <0.05 are marked with bold text.

		Autistic traits			
**Covariates in the model**		**No autistic traits, n (% of total)**	**Autistic traits, n (% of total)**	**aOR (95% CI)**	**P-value**
**Age 3 years**					
Prenatal ASM exposure due to epilepsy	No	16,395 (91)	1,635 (9)	**2.50 (1.30 – 5.10)**	**0.012**
	Yes	46 (84)	9 (16)		
Periconceptional folic acid use[1]	No	1,974 (88)	271 (12)	**0.71 (0.62 – 0.82)**	**<0.001**
	Yes	14,467 (91)	1,373 (9)		
Dietary folate intake (µg/day)	Mean (SD)	274.9 (96.6)	285.7 (122.0)	**1.00 (1.00 – 1.00)**	**<0.001**
Folic acid supplement dose (µg/day)	Mean (SD)	276.0 (275.5)	257.8 (275.0)	**1.00 (1.00 – 1.00)**	**0.037**
PRS of low folate[2]	Mean (SD)	-5.45e-05 (1.18e-03)	-4.66e-05 (1.19e-03)	1.00 (0.96 - 1.10)	0.800
*Prenatal ASM exposure * PRS of low folate[3]*				*1.50 (0.54 - 4.10)*	*0.440*
**Age 8 years**					
Prenatal ASM exposure due to epilepsy	No	13,626 (98)	231 (2)	**7.00 (2.10 – 23.00)**	**0.002**
	Yes	31 (91)	<5		
Periconceptional folic acid use[1]	No	1,613 (97)	49 (3)	**0.52 (0.38 – 0.72)**	**<0.001**
	Yes	12,044 (99)	185 (2)		
Dietary folate intake (µg/day)	Mean (SD)	275.5 (98.1)	277.1 (116.6)	1.00 (1.00 – 1.00)	0.820
Folic acid supplement dose (µg/day)	Mean (SD)	273.5 (269.3)	256.2 (387.2)	1.00 (1.00 – 1.00)	0.530
PRS of low folate[2]	Mean (SD)	-5.52e-05 (1.19e-03)	-4.88e-05 (1.09e-03)	1.00 (0.91 - 1.10)	0.900
*Prenatal ASM exposure * PRS of low folate[3]*				*1.40 (0.40 - 4.90)*	*0.610*
**Age 3 years**					
Prenatal ASM exposure due to epilepsy	No	16,395 (91)	1,635 (9)	**2.70 (1.00 – 7.00)**	**0.045**
	Yes	46 (84)	9 (16)		
Periconceptional folic acid use[1]	No	1,974 (88)	271 (12)	**0.71 (0.62 – 0.82)**	**<0.001**
	Yes	14,467 (91)	1,373 (9)		
Dietary folate intake (µg/day)	Mean (SD)	274.9 (96.6)	285.7 (122.0)	**1.00 (1.00 – 1.00)**	**<0.001**
Folic acid supplement dose (µg/day)	Mean (SD)	276.0 (275.5)	257.8 (275.0)	**1.00 (1.00 – 1.00)**	**0.046**
Maternal rs1801133 genotype	CC	8,221 (91)	799 (9)	1.10 (0.96 – 1.20)	0.260
	CT/TT	8,220 (91)	845 (9)		
*Prenatal ASM exposure * rs1801133 genotype[4]*				*0.64 (0.16 – 2.60)*	*0.543*

Autistic traits

**Age 8 years**					
Prenatal ASM exposure due to epilepsy	No	13,626 (98)	231 (2)	3.80 (0.52 – 27.00)	0.190
	Yes	31 (91)	<5		
Periconceptional folic acid use[1]	No	1,613 (97)	49 (3)	**0.52 (0.38 – 0.71)**	**<0.001**
	Yes	12,044 (99)	185 (2)		
Dietary folate intake (µg/day)	Mean (SD)	275.5 (98.1)	277.1 (116.6)	1.00 (1.00 – 1.00)	0.770
Folic acid supplement dose (µg/day)	Mean (SD)	273.5 (269.3)	256.2 (387.2)	1.00 (1.00 – 1.00)	0.500
Maternal rs1801133 genotype	CC	6,815 (99)	105 (2)	1.20 (0.94 – 1.60)	0.150
	CT/TT	6,842 (98)	129 (2)		
*Prenatal ASM exposure * rs1801133 genotype*[4]				*2.70 (0.25 – 29.00)*	*0.420*

[1] Any use during the period from four weeks before the pregnancy and the first trimester
[2] aOR is given as change per 0.001 unit of the PRS
[3] Interaction between prenatal ASM exposure due to maternal epilepsy and PRS of low folate concentrations. aOR is given as change per 0.001 unit of the PRS
[4] Interaction between prenatal ASM exposure due to maternal epilepsy and maternal rs1801133 genotype

ASM-exposed children of women with epilepsy were compared to ASM-unexposed children by using logistic regression models. Each age group was examined separately. Covariates in the adjusted models: periconceptional folic acid supplement intake (any intake during gestation week -4 to 12), dietary folate intake (µg/day), and folic acid supplement dose (µg/day).

Abbreviations: ASM, antiseizure medication; aOR, adjusted odds ratio; CI, confidence interval; NA, not applicable; NE, not estimable; PRS, polygenic risk score.

Table 5. Adjusted odds ratio (aOR) of language impairment in children of women with antiseizure medication (ASM)-untreated epilepsy compared to children of women without epilepsy stratified by maternal rs1801133 genotype.

Maternal rs1801133 genotype	Maternal ASM-untreated epilepsy	Child with language impairment, n (% of total)[1]		Crude OR (95% CI)	aOR (95% CI)
		No	Yes		
**Age 1.5 years**					
CC	No	12,272 (90)	1,296 (10)	1.00	1.00
	Yes	46 (94)	<5	0.62 (0.19 – 2.01)	0.74 (0.22 – 2.44)
CT/TT	No	12,325 (90)	1,319 (10)	1.00	1.00
	Yes	39 (87)	6 (13)	1.44 (0.62 – 3.35)	1.63 (0.64 – 4.19)
**Age 3 years**					
CC	No	10,393 (94)	659 (6)	1.00	1.00
	Yes	41 (95)	<5	0.77 (0.18 – 3.22)	0.98 (0.23 – 4.13)
CT/TT	No	10,372 (93)	759 (7)	1.00	1.00
	Yes	35 (95)	<5	0.78 (0.19 – 3.28)	0.98 (0.23 – 4.18)
**Age 5 years**					
CC	No	6,573 (80)	1,668 (20)	1.00	1.00
	Yes	15 (63)	9 (38)	2.36 (0.99 – 5.64)	**2.99 (1.18 – 7.54)**
CT/TT	No	6,523 (79)	1,725 (21)	1.00	1.00
	Yes	22 (82)	5 (19)	0.86 (0.33 – 2.21)	0.83 (0.29 – 2.34)
**Age 8 years**					
CC	No	6,597 (82)	1,459 (18)	1.00	1.00
	Yes	25 (76)	8 (24)	1.45 (0.64 – 3.26)	1.76 (0.76 – 4.10)
CT/TT	No	6,661 (82)	1,446 (18)	1.00	1.00
	Yes	22 (79)	6 (21)	1.26 (0.52 – 3.03)	1.47 (0.60 – 3.59)

[1] Crude numbers

Children of women with ASM-untreated epilepsy were compared to children of women without epilepsy stratified for maternal rs1801133 genotype by using multiple logistic regression models. Each age group was examined separately. Covariates in the adjusted models: periconceptional folic acid supplement intake (any intake during gestation week -4 to 12), dietary folate intake (µg/day), and folic acid supplement dose (µg/day).

Abbreviations: ASM, antiseizure medication; aOR, adjusted odds ratio; CI, confidence interval.

Table 6. Adjusted odds ratio (aOR) of autistic traits in children of women with antiseizure medication (ASM)-untreated epilepsy compared to children of women without epilepsy stratified by maternal rs1801133 genotype.

Maternal rs1801133 genotype	Maternal ASM-untreated epilepsy	Child with autistic traits, n (% of total)[1]		Crude OR (95% CI)	aOR (95% CI)
		No	Yes		
**Age 3 years**					
CC	No	9,829 (91)	1,001 (9)	1.00	1.00
	Yes	39 (93)	<5	0.76 (0.23 – 2.48)	0.93 (0.28 – 3.08)
CT/TT	No	9,854 (90)	1,059 (10)	1.00	1.00
	Yes	34 (94)	<5	0.55 (0.13 – 2.30)	0.00 (0.00 – 0.00)
**Age 8 years**					
CC	No	7,804 (98)	126 (2)	1.00	1.00
	Yes	31 (94)	<5	4.00 (0.94 – 16.97)	**4.92 (1.12 – 21.62)**
CT/TT	No	7,866 (98)	150 (2)	1.00	1.00
	Yes	28 (100)	0 (0)	0.00 (0.00 – 0.00)	0.00 (0.00 – 0.00)

[1] Crude numbers

Children of women with ASM-untreated epilepsy were compared to children of women without epilepsy stratified for maternal rs1801133 genotype by using multiple logistic regression models. Each age group was examined separately. Covariates in the adjusted models: periconceptional folic acid supplement intake (any intake during gestation week -4 to 12), dietary folate intake (µg/day), and folic acid supplement dose (µg/day).

Abbreviations: ASM, antiseizure medication; aOR, adjusted odds ratio; CI, confidence interval.

Table 7. Adjusted odds ratio (aOR) of language impairment in children of women with maternal rs1801133 genotype CT/TT compared to genotype CC within each of the three study groups.

Study group	Maternal rs1801133 genotype	Child with language impairment, n (% of total)[1]		Crude OR (95% CI)	aOR (95% CI)
		No	Yes		
**Children of women with ASM-treated epilepsy**	**Age 1.5 years**				
	CC	26 (84)	5 (16)	1.00	1.00
	CT/TT	35 (85)	6 (15)	0.89 (0.21 – 3.72)	0.42 (0.05 – 3.75)
	**Age 3 years**				
	CC	27 (100)	0 (0)	1.00	1.00
	CT/TT	28 (85)	5 (15)	NE	NE
	**Age 5 years**				
	CC	8 (57)	6 (43)	1.00	1.00
	CT/TT	15 (63)	9 (38)	0.80 (0.20 – 3.26)	0.73 (0.15 – 3.49)
	**Age 8 years**				
	CC	12 (63)	7 (37)	1.00	1.00
	CT/TT	10 (63)	6 (38)	1.03 (0.25 – 4.30)	0.48 (0.07 – 3.26)
**Children of women with ASM-untreated epilepsy**	**Age 1.5 years**				
	CC	46 (94)	<5	1.00	1.00
	CT/TT	39 (87)	6 (13)	2.36 (0.55 – 10.14)	3.28 (0.57 – 18.96)
	**Age 3 years**				
	CC	41 (95)	<5	1.00	1.00
	CT/TT	35 (95)	<5	1.17 (0.15 – 9.00)	1.11 (0.12 – 10.29)
	**Age 5 years**				
	CC	15 (63)	9 (38)	1.00	1.00
	CT/TT	22 (82)	5 (19)	0.38 (0.10 – 1.38)	0.29 (0.07 – 1.21)
	**Age 8 years**				
	CC	25 (76)	8 (24)	1.00	1.00
	CT/TT	22 (79)	6 (21)	0.85 (0.25 – 2.85)	1.05 (0.30 – 3.70)
**Children of women without epilepsy**	**Age 1.5 years**				
	CC	12,272 (90)	1,296 (10)	1.00	1.00
	CT/TT	12,325 (90)	1,319 (10)	1.01 (0.93 – 1.10)	0.98 (0.90 – 1.08)
	**Age 3 years**				
	CC	10,393 (94)	659 (6)	1.00	1.00
	CT/TT	10,372 (93)	759 (7)	**1.15 (1.03 – 1.29)**	**1.18 (1.05 – 1.34)**
	**Age 5 years**				
	CC	6,573 (80)	1,668 (20)	1.00	1.00
	CT/TT	6,523 (79)	1,725 (21)	1.04 (0.96 – 1.13)	1.05 (0.96 – 1.14)
	**Age 8 years**				
	CC	6,597 (82)	1,459 (18)	1.00	1.00
	CT/TT	6,661 (82)	1,446 (18)	0.98 (0.90 – 1.07)	0.99 (0.90 – 1.08)

[1] Crude numbers

Children of women with maternal rs1801133 genotype CT/TT were compared to children of women with genotype CC within each of the three study groups by using multiple logistic regression models. Each age group was examined separately. Covariates in the adjusted models: periconceptional folic acid supplement intake (any intake during gestation week -4 to 12), dietary folate intake (μg/day), and folic acid supplement dose (μg/day).

Abbreviations: ASM, antiseizure medication; aOR, adjusted odds ratio; CI, confidence interval; NE, not estimable.

Table 8. Adjusted odds ratio (aOR) of autistic traits in children of women with maternal rs1801133 genotype CT/TT compared to genotype CC within each of the three study groups.

Study group	Maternal rs1801133 genotype	Child with autistic traits, n (% of total)[1]		Crude OR (95% CI)	aOR (95% CI)
		No	Yes		
Children of women with ASM-treated epilepsy	**Age 3 years**				
	CC	21 (78)	6 (22)	1.00	1.00
	CT/TT	27 (82)	6 (18)	0.78 (0.20 – 2.96)	0.59 (0.10 – 3.61)
	**Age 8 years**				
	CC	18 (95)	<5	1.00	1.00
	CT/TT	14 (88)	<5	2.57 (0.19 – 34.02)	NE
Children of women with ASM-untreated epilepsy	**Age 3 years**				
	CC	39 (93)	<5	1.00	1.00
	CT/TT	34 (94)	<5	0.76 (0.12 – 4.99)	0.00 (0.00 – 0.00)
	**Age 8 years**				
	CC	31 (94)	<5	1.00	1.00
	CT/TT	28 (100)	0 (0)	0.00 (0.00 – 0.00)	0.00 (0.00 – 0.00)
Children of women without epilepsy	**Age 3 years**				
	CC	9,829 (91)	1,001 (9)	1.00	1.00
	CT/TT	9,854 (90)	1,059 (10)	1.06 (0.96 – 1.16)	1.07 (0.96 – 1.18)
	**Age 8 years**				
	CC	7,804 (98)	126 (2)	1.00	1.00
	CT/TT	7,866 (98)	150 (2)	1.18 (0.93 – 1.50)	1.24 (0.95 – 1.61)

[1] Crude numbers

Children of women with maternal rs1801133 genotype CT/TT were compared to children of women with genotype CC within each of the three study groups by using multiple logistic regression models. Each age group was examined separately. Covariates in the adjusted models: periconceptional folic acid supplement intake (any intake during gestation week -4 to 12), dietary folate intake (µg/day), and folic acid supplement dose (µg/day).

Abbreviations: ASM, antiseizure medication; aOR, adjusted odds ratio; CI, confidence interval; NE, not estimable.

Table 9. Number of antiseizure medication (ASM)-exposed children of women with epilepsy with and without language impairment and autistic traits stratified by type of ASM exposure in monotherapy and maternal rs1801133 genotype.

Study group	Maternal rs1801133 genotype	Child with language impairment, n (% of total)		Child with autistic traits, n (% of total)	
		No	Yes	No	Yes
Valproate monotherapy exposure	**Age 1.5 years**				
	CC	1 (33)	2 (67)		
	CT/TT	5 (83)	1 (17)		
	**Age 3 years**				
	CC	2 (100)	0 (0)	1 (50)	1 (50)
	CT/TT	4 (80)	1 (20)	4 (80)	1 (20)
	**Age 5 years**				
	CC	0 (0)	2 (100)		
	CT/TT	2 (67)	1 (33)		
	**Age 8 years**				
	CC	0 (0)	1 (100)	1 (100)	0 (0)
	CT/TT	2 (67)	1 (33)	2 (67)	1 (33)
Carbamazepine monotherapy exposure	**Age 1.5 years**				
	CC	6 (100)	0 (0)		
	CT/TT	8 (80)	2 (20)		
	**Age 3 years**				
	CC	5 (100)	0 (0)	4 (80)	1 (20)
	CT/TT	8 (89)	1 (11)	8 (89)	1 (11)
	**Age 5 years**				
	CC	3 (100)	0 (0)		
	CT/TT	4 (67)	2 (33)		
	**Age 8 years**				
	CC	4 (100)	0 (0)	4 (100)	0 (0)
	CT/TT	2 (40)	3 (60)	4 (80)	1 (20)
Lamotrigine monotherapy exposure	**Age 1.5 years**				
	CC	14 (93)	1 (7)		
	CT/TT	9 (90)	1 (10)		
	**Age 3 years**				
	CC	14 (100)	0 (0)	11 (79)	3 (21)
	CT/TT	7 (88)	1 (13)	6 (75)	2 (25)
	**Age 5 years**				
	CC	4 (57)	3 (43)		
	CT/TT	3 (60)	2 (40)		
	**Age 8 years**				
	CC	5 (63)	3 (38)	8 (100)	0 (0)
	CT/TT	3 (75)	1 (25)	4 (100)	0 (0)
